# TP53 mutation prevalence in normal airway epithelium as a biomarker for lung cancer risk

**DOI:** 10.1186/s12885-023-11266-7

**Published:** 2023-08-23

**Authors:** Daniel J. Craig, Erin L. Crawford, Heidi Chen, Eric L. Grogan, Steven A. Deppen, Thomas Morrison, Sanja L. Antic, Pierre P. Massion, James C. Willey

**Affiliations:** 1 University of Toledo College of Medicine, 3000 Arlington Ave, OH 43614 Toledo, USA; 2 Vanderbilt University Medical Center, 1301 Medical Center Dr., TN 37232 Nashville, USA; 3 Tennessee Valley VA Healthcare System, 1310 24Th Avenue South, Nashville, TN 37212 USA; 4 Accugenomics Inc, 1410 Commonwealth Dr #105, Wilmington, NC 28403 USA

**Keywords:** Lung cancer prevention, Lung cancer early detection, Biomarker, Next generation sequencing, TP53, Lung nodule risk classification

## Abstract

**Background:**

There is a need for biomarkers that improve accuracy compared with current demographic risk indices to detect individuals at the highest lung cancer risk. Improved risk determination will enable more effective lung cancer screening and better stratification of lung nodules into high or low-risk category. We previously reported discovery of a biomarker for lung cancer risk characterized by increased prevalence of TP53 somatic mutations in airway epithelial cells (AEC). Here we present results from a validation study in an independent retrospective case–control cohort.

**Methods:**

Targeted next generation sequencing was used to identify mutations within three TP53 exons spanning 193 base pairs in AEC genomic DNA.

**Results:**

TP53 mutation prevalence was associated with cancer status (*P* < 0.001). The lung cancer detection receiver operator characteristic (ROC) area under the curve (AUC) for the TP53 biomarker was 0.845 (95% confidence limits 0.749–0.942). In contrast, TP53 mutation prevalence was not significantly associated with age or smoking pack-years. The combination of TP53 mutation prevalence with PLCO_M2012_ risk score had an ROC AUC of 0.916 (0.846–0.986) and this was significantly higher than that for either factor alone (*P* < 0.03).

**Conclusions:**

These results support the validity of the TP53 mutation prevalence biomarker and justify taking additional steps to assess this biomarker in AEC specimens from a prospective cohort and in matched nasal brushing specimens as a potential non-invasive surrogate specimen.

**Supplementary Information:**

The online version contains supplementary material available at 10.1186/s12885-023-11266-7.

## Introduction

Lung cancer causes more deaths worldwide than any other cancer [1]. The National Lung Screening Trial (NLST) and the NELSON trial each demonstrated that early detection through low dose computed tomography (LDCT) screening reduces lung cancer mortality by at least 20% compared to chest x-ray screening [2–4]. Based on the strength of data from these and other studies, the latest guidelines from the United States Preventative Services Task Force (USPSTF) recommend expansion of LDCT screening eligibility to individuals 50–80 years of age with at least a 20 pack-year smoking history who quit < 15 years prior.

In spite of these important advances, there remains an opportunity to better stratify individuals for LDCT screening based on biomarker risk criteria that supplement demographic risk criteria. For example, a large fraction of lung cancers are found in individuals who do not meet even the expanded LDCT screening threshold criteria [5], including those younger than 50. This younger group comprised 28% of lung cancer cases in one study [6]. Further, roughly 25% of all lung cancer cases worldwide occur in never smokers and this proportion will continue to increase due to smoking cessation and prevention efforts [7, 8].

Another opportunity is to reduce the large number of false positive findings associated with LDCT screening. Specifically, each early lung cancer first appears on a chest CT as an indeterminate pulmonary nodule (IPN) (defined as a well-defined, non-calcified, nodule in the lung less than 3 cm in size), but > 95% of nodules identified by screening are the result of a benign process, such as scar tissue, an infection, or non-infectious inflammatory process [9]. Based on nodule size, shape, and location characteristics, combined with patient age and smoking history, cancer risk for each nodule may be designated as low, intermediate, or high (e.g. < 15%, 15–75%, or > 75%, respectively) [10]. For example, regardless of lung cancer risk based on smoking history and age of the individual, the probability of malignancy is less than 1% for all nodules smaller than 6 mm, and 1% to 2% for nodules 6 mm to 8 mm [11]. The American College of Radiology [12], the National Comprehensive Cancer Network (NCCN) [13], the American College of Chest Physicians (ACCP) [10], and other consensus groups agree that low-risk nodules may be safely followed with an interval CT scan (e.g., three to six months) to assess for growth, while high-risk nodules should be subjected to expedited diagnosis and/or surgical removal. However, intermediate nodules represent about 1/3 of all nodules and present a significant clinical challenge. Specifically, intermediate nodules are associated with the highest rate of diagnostic errors, in part because there is no clear strategy for their management [14]. Thus, a pressing unmet need is a biomarker that will reliably move intermediate-risk nodules to high or low-risk [15].

A prevalent strategy to more accurately identify individuals at risk for cancer or to detect early-stage cancer is to develop biomarkers based on inherited (i.e., germ cell) and/or acquired (i.e., somatic cell) genetic risk determinants [16–24]. In an application of this strategy, we used an optimized targeted NGS method [21, 25, 26] in a discovery study to measure driver gene mutations, including those with low variant allele frequency (VAF) (mutations between 0.01–1.0% VAF), in grossly normal airway epithelial cells (AEC) [21, 26]. We discovered that TP53 mutations at known lung cancer hotspot sites within three TP53 exons 5–7 were significantly more prevalent in AEC specimens from lung cancer cases compared to non-cancer controls [21]. Here, we evaluated this TP53 mutation biomarker in a larger independent retrospective case–control cohort using AEC specimen DNA from lung cancer and non-cancer subjects.

## Methods

### Study cohort enrollment and characterization

Subjects undergoing bronchoscopy for clinical purposes provided informed consent and were enrolled into one of two approved research protocols at Vanderbilt University Medical Center (VUMC) (Table 1).

**Table 1:** Patient Demographics

Sample #	Patient ID	Cancer Status	PY^c^	Sex	Age^d^	Smoking Status	Histology	Brock Risk Score	TP53 Mutation Prevalence^g^	Protocol ID^h^
								Risk Score (× 100)		
1	7047	NC^a^	25	M	57	Former	NC	0.86	0.000	0398
2	7768	NC	20	F	52	Current	NC	1.07	0.005	0398
3	8115	NC	36	M	56	Former	NC	0.66	0.000	1078
4	9277	NC	20	F	60	Former	NC	0.94	0.000	1078
5	9680	NC	31	F	63	Former	NC	0.97	0.010	1078
6	12,028	NC	14.5	F	50	Current	NC	0.4	0.015	0398
7	12,318	NC	6	M	71	Former	NC	0.68	0.005	0398
8	7871	NC	45	M	69	Former	NC	9.31	0.020	0398
9	8027	NC	78	F	55	Current	NC	5.88	0.026	1078
10	8182	NC	110	M	61	Former	NC	11.07	0.005	1078
11	8202	NC	78	M	66	Current	NC	7.05	0.010	1078
12	8356	NC	42	F	72	Former	NC	6.15	0.005	1078
13	9299	NC	75	M	64	Current	NC	5.26	0.000	1078
14	11,858	NC	20	M	74	Former	NC	6.59	0.000	0398
15	7878	NC	30	F	58	Current	NC	2.15	0.005	1078
16	7319	NC	0	M	28	Never	NC	0.04	0.005	0398
17	7282	NC	0	F	33	Never	NC	0.08	0.000	0398
18	7291	NC	0	M	38	Never	NC	0.1	0.010	0398
19	10,660	NC	0	F	41	Never	NC	0.14	0.000	0398
20	9425	NC	66	F	70	Current	NC	7.16	0.015	1078
21	9022	NC	51	M	60	Former	NC	4.16	0.020	1078
22	8978	NC	51	M	65	Current	NC	8.48	0.000	1078
23	12,444	NC	60	F	68	Current	NC	11.28	Not measured	1078
38	11,583	NC	39	M	63	Former	NC	1.38	0.005	0398
39	9420	NC	62.5	F	59	Former	NC	2.1	0.000	1078
40	7769	NC	0	M	70	Never	NC	1.65	0.000	0398
41	11,202	NC	0	F	52	Never	NC	0.55	0.000	0398
42	11,917	NC	0	F	81	Never	NC	6.93	0.000	0398
43	15,667	NC	0	M	57	Never	NC	1.18	0.005	0398
44	7979	NC	62	F	61	Former	NC	1.73	0.000	1078
24	6359	CA^b^	44	M	68	Former	SQ^e^	8.55	0.041	0398
25	11,143	CA	47	M	65	Current	AD^f^	16.79	0.005	0398
26	11,761	CA	78	M	49	Current	AD	5.25	0.026	0398
27	11,841	CA	72	M	51	Current	AD	2.19	0.026	0398
28	11,704	CA	72	F	61	Current	SQ	14.6	0.031	0398
29	7481	CA	150	M	78	Former	SQ	14.95	0.005	0398
30	8841	CA	185.5	M	76	Former	SQ	22.88	0.041	0398
31	7725	CA	60	M	79	Current	SQ	25.61	0.041	0398
32	8358	CA	50	M	60	Current	SQ	9.42	0.056	0398
33	8439	CA	92	M	62	Former	SQ	5.3	0.051	0398
34	7801	CA	25	F	65	Current	SQ	2.65	0.036	0398
35	7509	CA	100	M	68	Current	SQ	13.74	0.026	0398
36	7492	CA	40	M	63	Current	SQ	4.29	0.020	0398
37	8340	CA	108	M	64	Former	SQ	9.93	0.031	0398
45	12,847	CA	15	F	59	Former	AD	2.28	0.000	0398
46	3857	CA	47	F	67	Former	AD	7.17	0.010	0398
47	3955	CA	88	F	58	Current	AD	14	0.031	0398
48	6077	CA	88	F	58	Current	AD	14.37	0.010	0398
49	7757	CA	45	M	74	Former	AD	11.15	0.097	0398
50	7781	CA	99.9	F	60	Former	AD	4.95	0.031	0398
51	7789	CA	30	F	77	Former	AD	12.04	0.005	0398
52	7862	CA	74	M	68	Former	AD	4.42	0.010	0398
53	9517	CA	40	F	61	Former	AD	3.09	0.000	0398
54	9641	CA	50	F	64	Current	AD	7.31	0.031	0398
55	9707	CA	60	F	77	Former	AD	11.22	0.005	0398
56	9798	CA	47	F	69	Former	AD	8.52	0.056	0398
57	11,246	CA	96	F	65	Current	AD	7.35	0.010	0398
58	12,911	CA	15	M	72	Former	AD	5.3	0.046	0398
59	14,611	CA	60	F	69	Current	AD	10.43	0.005	0398
60	14,813	CA	61.5	M	59	Former	AD	6.24	0.031	0398

^a^ Noncancer

^b^ Lung cancer

^c^ Pack-years

^d^Age at Collection

^e^ Squamous cell carcinoma

^f^ Adenocarcinoma

^g^ TP53 mutations/193 bp targeted/subject

^h^ See Methods Sect. 0398—Molecular Predictors of Lung Cancer Behavior, 1078—Nashville Lung Screening Trial

The first VUMC protocol (Molecular Predictors of Lung Cancer Behavior- 0398) included subjects aged 18–80 years with IPN between 6 and 30 mm in the largest axial diameter detected incidentally or through screening.

The second VUMC protocol (Nashville Lung Screening Trial-1078) included subjects aged 55 years or older with screening detected nodules and a > / = 25 pack-year smoking history. Current and former smokers (quit < 15 years prior) were enrolled. In each protocol, cases were subjects with lung cancer confirmed through positive biopsy, and controls were subjects confirmed to not have cancer through negative biopsies and/or 2-year longitudinal imaging follow-up with no sign of growth.

AEC specimens were collected by bronchoscopic brush biopsy of grossly normal (not overtly metaplastic or abnormal-appearing to the trained pulmonologist) airway from 30 lung cancer (CA) cases all of whom had a smoking history, and 30 non-cancer (NC) controls. Brushings were taken from the opposite lung or a region distant from known or suspected disease using cytology brushes (Cook Medical BCB-5–120-3-S). AEC were collected by rotating the brush while completing 20–30 brush strokes. Brushes were clipped into empty tubes, immediately frozen on dry ice, then stored at -80 °C until DNA extraction.

The PLCO_M2012_ for lung cancer risk score was calculated for each subject based on demographic characteristics as previously described [27].

### DNA extraction

Genomic DNA (gDNA) was extracted from each AEC specimen at Vanderbilt using the DNeasy Blood and Tissue Kit (Qiagen, Hilden, Germany) according to manufacturer protocol and assessed for purity using NanoDrop. Aliquots of genomic DNA extracted from the AEC specimens were de-identified and blinded by VUMC and provided to the University of Toledo (UToledo) through an approved material transfer agreement.

### Targeted NGS method

A quality-controlled targeted NGS method [21, 26] was used to measure mutations in gDNA extracted from each AEC specimen at known lung cancer hotspot sites within TP53 exons 5–7 (designated TP53.5, TP53.6, TP53.7 in this study) spanning 193 bp.

### DNA quantification

The number of amplifiable gDNA copies present in each sample was quantified at UToledo using competitive polymerase chain reaction (PCR) amplification of a well-characterized genomic locus in the Secretoglobin, family 1A, member 1 gene (SCGB1A1), as previously described previously using SCGB1A1-q primers (Supplementary Table 1) and SCGB1A1 genomic DNA reagents (Accugenomics, Inc., Wilmington, NC) [28, 29].

### Target primer design

Primer sequences and priming strategy are depicted in Supplementary Table 1 and Supplementary Fig. 1. TP53 primers were designed to amplify both gDNA and complementary DNA (cDNA), although only gDNA was used in this study. An alien tag sequence (APEX) was added to the 5’ end of each primer as previously reported [29] to enable addition of barcode/sequencing adaptors in a downstream reaction. Barcode/adaptor primers comprise an Illumina P5/read 1 or P7/read 2 region, a 4-base variable pad region, a 10-base barcode region and a region complementary to the APEX tag sequence at the 5’ ends of the target primers (Supplementary Fig. 1). A complete list of barcodes is presented in Supplementary Table 2. Target-specific primer sequences were designed to optimize PCR efficiency, and primers were synthesized as standard, desalted oligos as a service at Integrated DNA Technologies (IDT, Coralville, IA). Barcode/adapter primers were synthesized using the IDT Ultramer platform due to the length of these primers (93–96 bases).

### Internal Standard (IS) design

Competitive synthetic DNA internal standard (IS) molecules for TP53 targets described above were designed, using methods previously described, to mimic each target area but with substituted dinucleotides approximately every 50 bases to enable bioinformatic separation of native template (NT) and IS reads following sequencing (details in Supplementary Methods). The IS for each target was included in each assay to control for technical sequencing error as described previously [21, 26].

### External Complexity Calibration Ladder (ECCL)

We designed a control for PCR amplicon library complexity using a known number of synthetic SCGB1A1 IS molecules to ensure that we reliably measured original genomic copies loaded. The design of the ECCL is provided in Supplementary Methods.

### Amplicon library generation

In order to maximize the number of genome copies loaded into the assay for each sample and thereby maximize the opportunity to detect low frequency variants in each target, a multiplex competitive PCR amplicon library was prepared for each AEC gDNA sample [21]. Conditions were optimized to minimize technical error during PCR, including use of Q5 HotStart High Fidelity DNA Polymerase that has a reported error frequency of 10^–6^ (New England Biolabs, Ipswich, MA) and minimization of PCR cycles in each of two rounds (details in Supplementary Methods). Each PCR reaction was prepared containing at least 50,000 genome equivalents of both AEC gDNA sample and ISM.

### Sequencing

The purified sequencing library was sent to the University of Michigan Advanced Genomics Core facility for Next Generation Sequencing on an Illumina NovaSeq 6000 SP flow cell with a 20% PhiX spike-in control.

### Analysis of NGS Data

FASTQ files were received from the University of Michigan Genomics core facility and processed using the Qiagen CLC Genomics Workbench software suite for quality-trimming, alignment, and variant calling. A modified IS reference genome was made in silico by concatenating each IS reference sequence as a separate contig to the end of the hg19 reference genome. Primer sequences and internal standard dinucleotide positions plus the nucleotides on their 5’ and 3’ sites, were excluded from variant analysis.

Spiking a known number of synthetic SCGB1A1 IS ladder molecules into each sample controlled for a) sample loading and b) down-sampling of sequencing reads from each library prep to represent the true number of molecules captured for each sample. This step satisfied the Poisson statistical criterion that each event (observed sequencing read) be independent (Supplementary Tables 3 and 4). Down-sampling was completed as follows: First, if the lowest diluted IS in the ladder yielded a minimum threshold number of reads, we inferred that at least 50,000 molecules of SCGB1A1 IS1 were captured. If SCGB1A1 IS represented at lower concentrations in the ECCL did not yield sufficient reads, the expected SCGB1A1 IS1 molecule number was adjusted down from 50,000 accordingly (Supplementary Table 3). Next, measured SCGB1A1 molecules in the sample (NT molecules) were calculated using the formula:

$$\frac{SCGB1A1\;NT\;reads}{SCGB1A1\;IS1\;reads}\times SCGB1A1\;IS1\;adjusted\;molecules$$

Finally, target NT molecules measured were calculated for each TP53 target using the formula:

$${}^{\frac{Target\;NT\;reads}{SCGB1A1\;NT\;reads}}\!\left/ \!{}_{SCGB1A1\;NT\;molecules}\right.$$

### Variant calling

The Basic Variant Detection tool in Qiagen CLC Genomics Workbench software was used to identify every single nucleotide variant (SNV) present in the patient sample NT reads as well as the IS reads for that patient sample. As described above, the methods used to synthesize the IS molecules for this study result in very low synthesis-error variant frequency (VAF < 0.0001%), a level sufficiently low that it would not confound analysis of biological mutations with VAF of 0.01% or higher that were the subject of this study. Thus, any variants in the synthetic IS spike-in measured with VAF > 0.01% resulted from technical error during library preparation or sequencing. As such, the variant allele frequency (VAF) measured for each type of transition/transversion at each base position in the IS enabled limit of blank (LOB) calculation. Poisson Exact Test (PET) was then used to determine significance of each detected NT variant relative to the LOB measurement in the IS for each type of mutation at each base position, as previously described [26]. A Bonferroni correction for false discovery was used based on the number of nucleotides assessed (193 bp) and the number of substitution mutations possible at each nucleotide position (*N* = 3). Further, to minimize potential analytical variation resulting from stochastic sampling, only mutations present in > 5 NT molecules measured were included.

### Variant annotation and hotspot analysis

Called variants were characterized for pathogenicity using publicly available databases including dbSNP, COSMIC, and FASMIC. Identification of known oncogenic hotspots and generation of corresponding figures were assessed using the cBioPortal for Cancer Genomics developed at Memorial Sloan Kettering (MSK) Cancer Center [30].

### Statistical analysis

#### Primary endpoint

The primary endpoint was to determine whether the diagnostic performance of the TP53 biomarker in this expanded case–control cohort was consistent with that reported in the discovery study [21]. This biomarker comprises the number of unique TP53 mutation clones in an AEC specimen obtained by bronchoscopy from each subject (TP53 mutation prevalence). In primary endpoint analysis, the biomarker was assessed for association with lung cancer risk based on the mean TP53 mutation prevalence in the 193 bp assessed in AEC among all cases (mutations/bp/30 subjects) vs all controls (mutations/bp/29 subjects) (Kruskal–Wallis), and the association of TP53 mutation prevalence with cancer diagnosis based on receiver operator characteristic (ROC) area under the curve (AUC).

### Secondary endpoint

Assessment for previously reported significant mutation enrichment among cases in a) tobacco smoke or age signatures, or b) with TP53 “hot-spots” was assessed with Kruskal–Wallis test using a Chi-square distribution.

### Exploratory endpoint

A combined biomarker (CBM) comprising the TP53 biomarker and the PLCO_M2012_ risk score was calculated using a logistic regression model with flexible functional forms, as previously described [31]. Comparison between the TP53 biomarker alone, PLCO_M2012_ alone, and the CBM was based on ROC analysis well as risk distribution based on sampling distribution of sample proportions ($\widehat{p}$) and Wilcoxon rank sum test with continuity correction [31].

## Results

Subject characteristics are presented in Table 1. Comparison of cases and controls with respect to key demographic characteristics are presented in Table 2. Of the specimens from 60 subjects studied, data were processed for all three TP53 exons in specimens from 59 subjects, including 30 cases and 29 controls. The specimen from one control (Subject 23) was excluded due to insufficient sequencing reads (Supplementary Table 4). For the remaining 59 subjects the sequencing yield was roughly 1 billion 2 × 150 paired-end reads with a 20% PhiX spike-in control. This resulted in ~ 26.3 million ± 4.3 million (range: 13.5 million – 41.4 million) reads for each sample representing a 25-30X sequencing depth for each target (Supplementary Table 4).

**Table 2:** Summary Demographic Characteristics of Cohort With TP53 Biomarker Data

Characteristic	N	NC^a^	CA^b^	*P* value	Test used
**Age**	59	58.8 ± 12.5	65.5 ± 7.6	**0.027**	Wilcoxon
**Gender**	59	14 Female / 15 Male	14 Female / 16 Male	0.902	Pearson
**Race**	59			0.281	Pearson
African-American		0	2		
Asian		1	0		
Caucasian		26	28		
Native American		1	0		
Unknown/Other		1	0		
**Smoking Status**	59			**0.007**	Wilcoxon
Current		8 (28%)	14 (47%)		
Former		13 (45%)	16 (53%)		
Never		8 (28%)	0 (0)		
**Cigarette Pack-years**	51	45.8 ± 26.1	68.0 ± 37.3	**0.031**	Wilcoxon

^a^ Noncancer

^b^ Lung cancer

### Diagnostic performance of the TP53 mutation biomarker

**Fig. 1:**
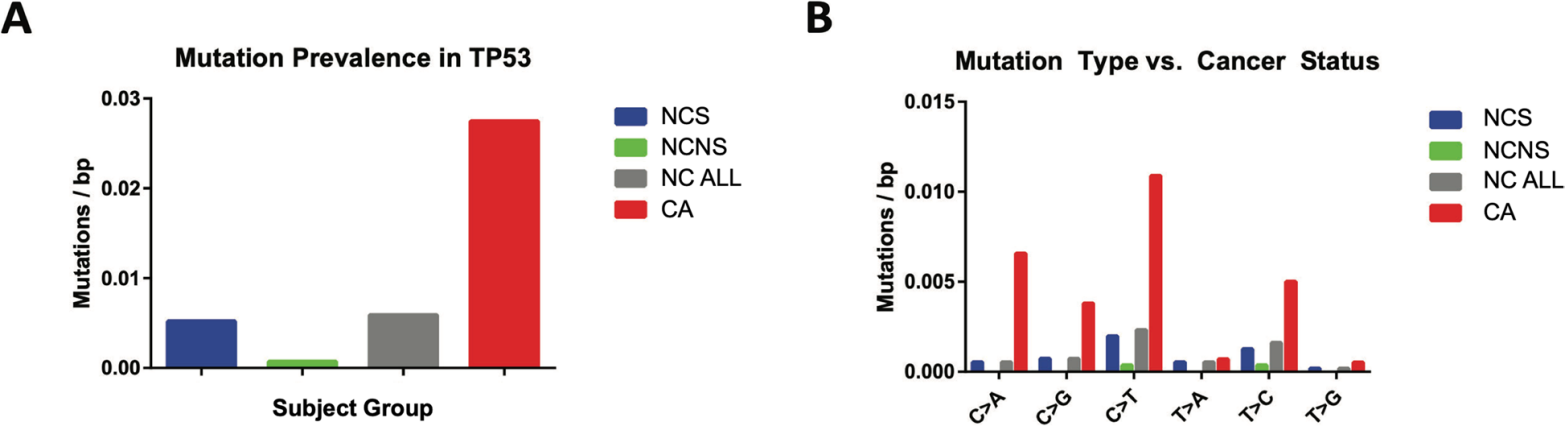
**a** Subject group-specific mean mutation prevalence (mutations/bp) in the targeted TP53 exon regions (spanning 193 bp) among control non-cancer smokers (NCS) (*N* = 21), control non-cancer non-smokers (NCNS) (*N* = 8), all controls (NC ALL) (*N* = 29) and cancer (CA) (*N* = 30) subjects. Prevalence in AEC specimens from CA subjects was significantly higher compared with NC ALL (*P* < 0.001) while there was no significant difference between NCS and NCNS (Kruskal–Wallis). **b** Assessment of TP53 mutation signature patterns as prevalence (mutations/bp) according to nucleotide transition type. After Bonferroni adjustment, there was a significantly higher prevalence of C > A (*P* = 0.001), C > T (*P* = 0.0013), and T > C (*P* = 0.0024) in CA compared with NC ALL (Kruskal–Wallis test using a Chi-square distribution)

A total of 192 unique biological TP53 mutations with VAF > 0.01% were observed within the targeted regions (193 bp) among specimens from the 59 subjects studied. All of these mutations were missense except for one silent mutation observed in a control subject (Supplementary Table 5). After un-blinding we determined that the mean TP53 mutation prevalence (mutations/bp) among AEC specimens from cases was significantly higher compared to controls (*P* < 0.001, Kruskal–Wallis) (Fig. 1a). Specifically, of the 192 mutations observed, there were 159 mutations among the cases (prevalence 0.027 mutations/bp) and 33 mutations among the controls, (prevalence 0.006 mutations/bp). Notably, TP53 mutation prevalence in AEC was not associated with smoking pack-years among the entire group (*N* = 59), the 30 cases, or the 29 controls (Table 3). There was also no association of TP53 mutation prevalence with age, though age range in this population was relatively restricted (Table 3). In this study there was not sufficient information to assess effect of reported race on association of the TP53 biomarker with lung cancer (Tables 2 and 3).

**Table 3:** TP53 Biomarker Association with Demographic Characteristics

Characteristic	TP53 Biomarker Assocation (*P* value)		
	All (*N* = 59)	Controls (*N* = 29)	Cases (*N* = 30)
**Age**	0.2	0.883	0.773
**Gender**	0.147	0.799	0.054
**Race**	0.655	0.554	1
**Pack-years**	0.46	0.18	0.838

### Tobacco signature TP53 mutation characteristics

Mutation substitution types with known cigarette smoke exposure association and phenotypic effects were significantly enriched among AEC specimens from cases and closely approximated the spectrum of TP53 mutations reported for lung cancer tissues (Fig. 1b) [32, 33]. Specifically, as observed in the discovery study nearly all of the TP53 mutations in cases were tobacco smoke signature (C > A) or age signature (C > T, T > C) mutations [21], [34]. After Bonferroni adjustment the difference between cases and controls was significant for each of these substitution types: C > A (*P* = 0.001), C > T (*P* = 0.0013), and T > C (*P* = 0.0024). Notably, the proportion of mutations at known TP53 hot-spot locations was significantly higher among cases compared to controls (Supplementary Fig. 2).

### ***Diagnostic accuracy of a CBM comprising the TP53 biomarker and the PLCO***_***M2012***_*** risk score compared with each biomarker alone***

A logistic regression model with flexible functional forms as previously described [31] was used to develop a CBM comprising the PLCO_M2012_ risk score continuous values (PRS.con) and the T53 biomarker continuous values (TP53.con) as:

$$\mathrm{Prob}\left\{\mathrm y=\right\}=\frac1{1+\exp(-\mathrm X\mathrm\beta},$$

where $$X\widehat\beta=-3.165853+29.31218\mathrm{PRS}.\mathrm{con}+0.5462315\mathrm{TP}53.\mathrm{con}$$ 

**Fig. 2:**
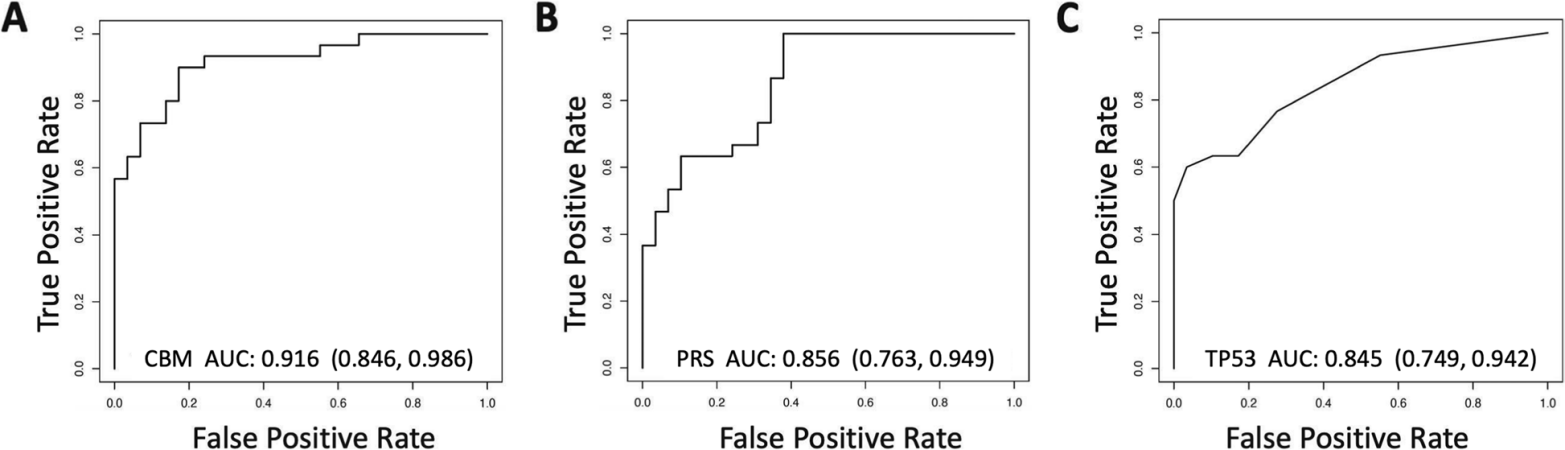
ROC curves with AUC (95% confidence limits) for **a** CBM comprising the PLCO_M2012_ Risk Score (PRS) + TP53, **b** PRS alone, and **c** TP53 biomarker alone. The AUC for the CBM was significantly higher (*P* = 0.03) compared to PRS or TP53 alone

**Fig. 3:**
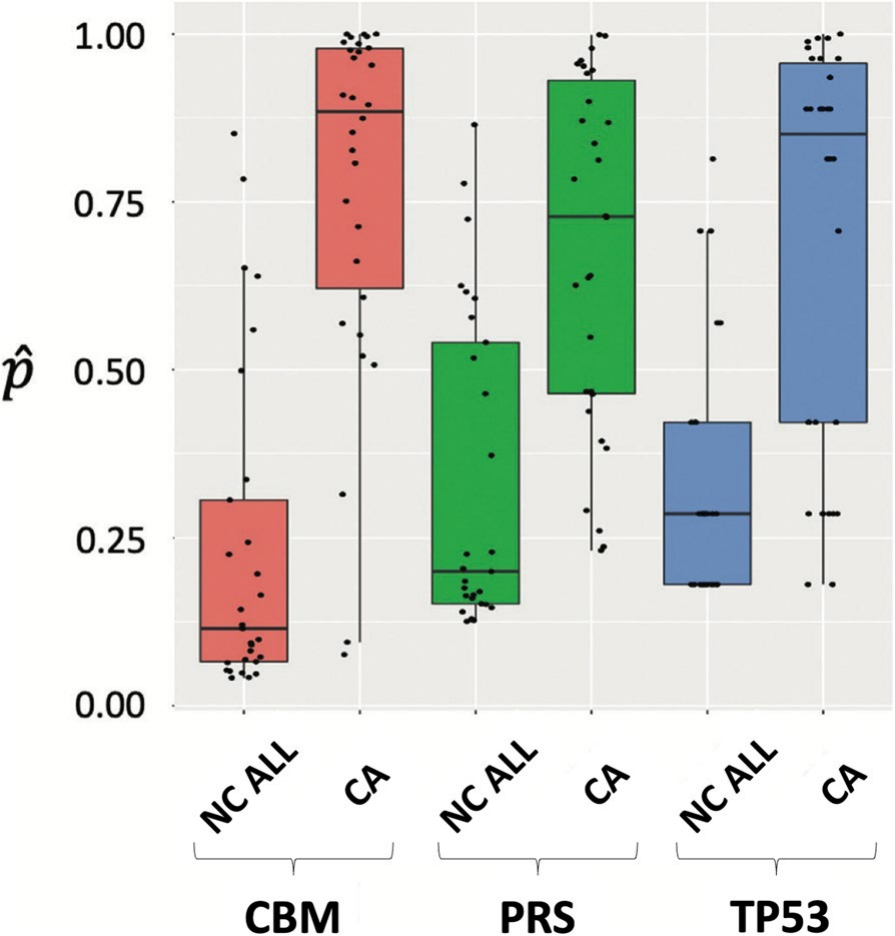
Probability distributions comparing NC ALL vs CA subjects for the CBM, the PLCO_M2012_ Risk Score (PRS) alone, and TP53 biomarker alone. The difference between NC ALL and CA was significant for the CBM (*P* = 1.436e-09), the PRS alone (*P* = 2.696e-06), and the TP53 biomarker alone (*P* = 3.856e-06)

Using the Youden optimal cutoff, the diagnostic accuracy (mean ROC AUC [± 95% confidence limits]) for a CBM comprising the T53 biomarker with the PLCO_M2012_ risk score was 0.916 [0.846–0.986] (Fig. 2a) and this was significantly higher than for the PLCO_M2012_ risk score alone (0.856 [0.763–0.949]) (*P* < 0.03) (Fig. 2b) or the TP53 biomarker alone (0.845 [0.749–0.942]) (*P* < 0.03) (Fig. 2c). As presented in Fig. 3, based on probability distribution analysis the difference between cases and controls was significant for the CBM (*P* < 0.001), the PLCO_M2012_ risk score alone (*P* < 0.001), and the TP53 biomarker alone (*P* < 0.001). As is evident, in comparison with the PLCO_M2012_ risk score alone or TP53 biomarker alone, for the CBM the distribution of intermediate risk nodules was more shifted to high or low risk.

## Discussion

This independent retrospective case–control cohort study confirmed our prior discovery that somatic TP53 mutations are significantly more prevalent in grossly normal AEC specimens from cases with lung nodules diagnosed as cancer compared with controls with lung nodules confirmed to be benign (Fig. 1a). Moreover, confirmation of our previous observation that these TP53 mutations represent tobacco-smoke signatures (Fig. 1b) and are primarily in hot-spot driver mutation sites (Supplementary Fig. 2) [21] further supports the conclusion that they are biological true positives.

Importantly, the TP53 biomarker was not associated with smoking pack-years (Table 3). This observation is consistent with our hypothesis that the TP53 biomarker measures the effect of hereditary susceptibility to lung cancer and, therefore, is independent and synergistic with risk conferred by cigarette smoke exposure. Specifically, it is hypothesized that AEC TP53 mutations and lung cancer each occur predominantly in those individuals with a heavy smoking history who also inherit a set of germ line variants that increase their risk for somatic mutations caused by exposure to cigarette smoke inhalation. Possible mechanisms for hereditary predisposition include sub-optimal DNA repair or antioxidant protection [18, 35, 36] and higher risk for nicotine addiction [36]. If this hypothesis is correct, the TP53 biomarker may serve as a summation biomarker for both hereditary risk as well as the degree of acquired risk from smoking and other forms of environmental exposure. For example, individuals with the same pack-year smoking history may acquire a different prevalence of TP53 mutations not only due to differences in hereditary risk, but also differences in cigarette smoking characteristics, such as brand of cigarettes, depth of inhalation, or the number of inhalations per cigarette, and/or a difference in inhalational exposure to other carcinogens, such as radon.

Based on ROC analysis, the TP53 biomarker at 100% specificity has a relatively high sensitivity (Fig. 2c). This is in contrast to demographic risk criteria such as the PLCO_M2012_ risk score that have moderately high specificity with 100% sensitivity, as observed in our cohort (Fig. 2b). This difference at least partly explains the observation that the CBM had significantly higher ROC AUC than either the TP53 biomarker or PLCO_M2012_ risk score alone (Fig. 2a), and better-identified nodule probability for malignancy (Fig. 3). In future studies, through collaboration with the EDRN, we plan to explore the combination of TP53 biomarker with other promising biomarkers [31, 35, 37–41].

## Conclusion

The strong performance of the TP53 biomarker in this independent cohort justifies additional validation studies, including analysis of TP53 mutation prevalence alone and in CBM in AEC specimens as well as nasal brush as potential non-invasive surrogate specimens from prospective cohorts. If validated, this biomarker may help achieve the key goals to reduce unnecessary invasive tests for benign nodules and reduce time to diagnosis for malignant nodules [31] as well as better stratify patients for lung cancer prevention trials and improve performance of annual LDCT screening. Specifically, use of this biomarker may enable selection of more optimal eligibility criteria and thereby reduce cost and false positive results associated with prevention trials and LDCT screening. Moreover, this biomarker may increase screening access for individuals who are at increased risk but do not meet current eligibility criteria based on demographic factors alone. The performance of this biomarker in different racial groups will require additional study.

### Supplementary Information

**Additional file 1.**

**Additional file 2.**

**Additional file 3.**

**Additional file 4.**

## Data Availability

All data generated in this will be made available upon reasonable request to the corresponding author. Biological samples have either been exhausted or limited amounts remain and, therefore, will not be made available. Sequencing data are available through the National Library of Medicine National Center for Biotechnology Information under SRA number PRJNA1001394 (https://www.ncbi.nlm.nih.gov/sra/PRJNA1001394).”
