# Supplementary figures and images for: TP53 mutation prevalence in normal airway epithelium as a biomarker for lung cancer risk
Source: BMC Cancer. 2023 Aug 23;23:783. doi: 10.1186/s12885-023-11266-7 (PMC10464352; doi:10.1186/s12885-023-11266-7)

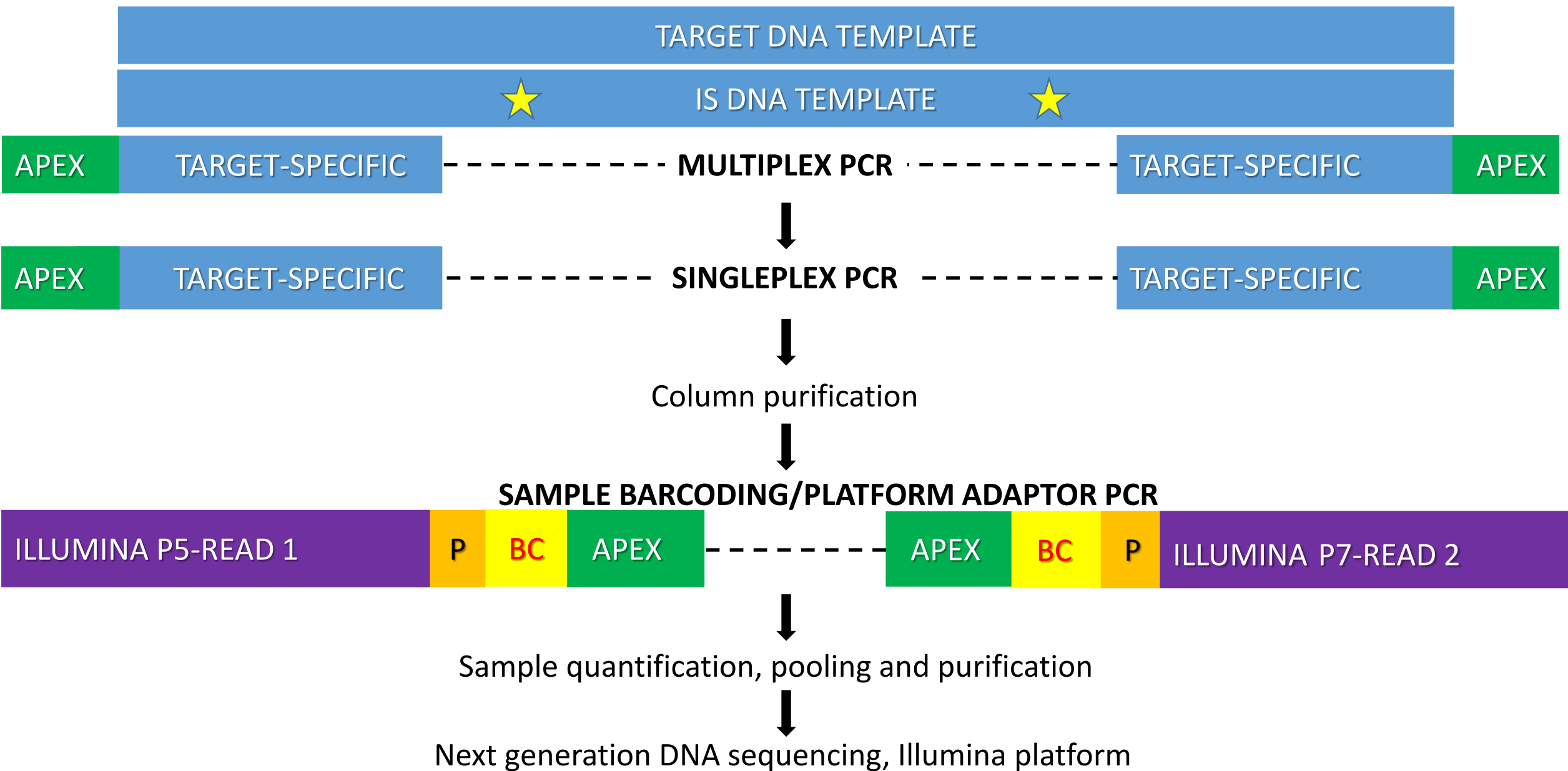

Supplement: Supplementary file 2 — Additional file 2. [file 12885_2023_11266_MOESM2_ESM.pdf]

**Case Subjects (CA): 159** hot-spot mutations (n=30)

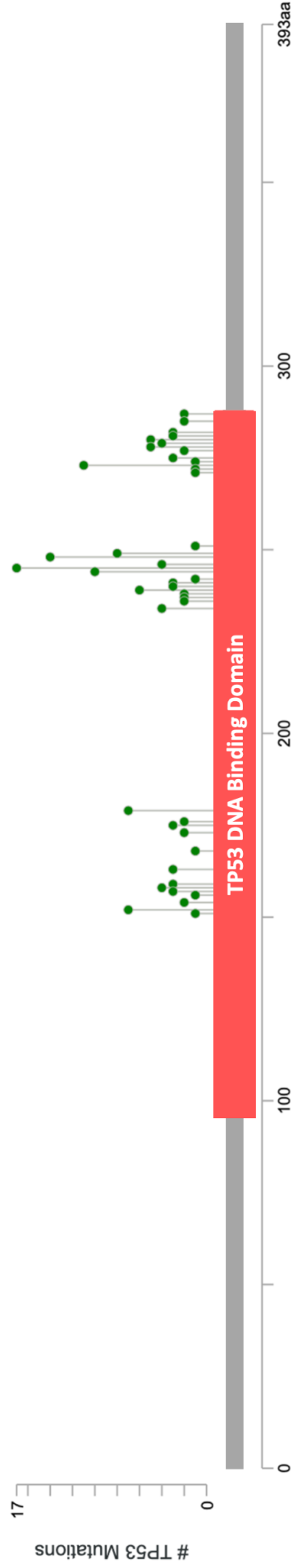

**Control Subjects (NC ALL): 33** hot-spot mutations (n=29)

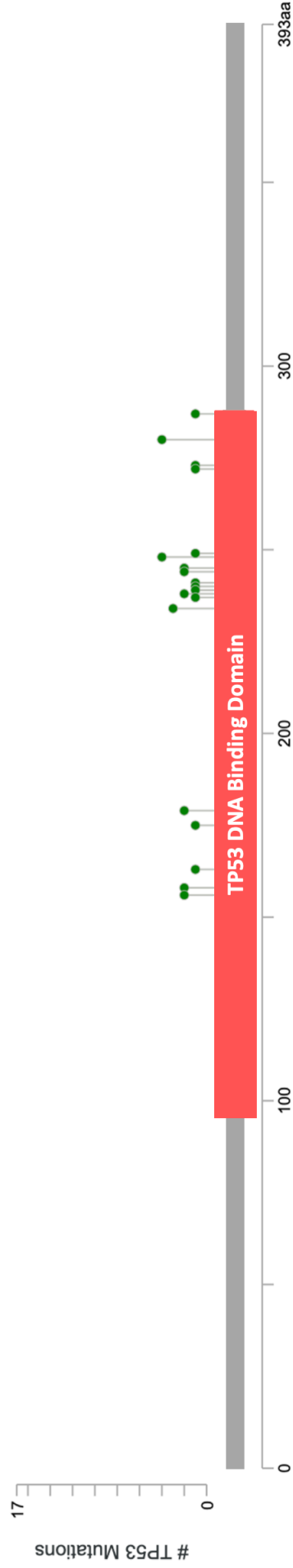

Supplement: Supplementary file 3 — Additional file 3. [file 12885_2023_11266_MOESM3_ESM.pdf]
